# Characterization of glutamate carboxypeptidase 2 orthologs in trematodes

**DOI:** 10.1186/s13071-022-05556-5

**Published:** 2022-12-20

**Authors:** Lucie Jedlickova, Kristyna Peterkova, Enoch Mensah Boateng, Lenka Ulrychova, Vojtech Vacek, Zsofia Kutil, Zhenze Jiang, Zora Novakova, Ivan Snajdr, Juan Kim, Anthony J. O’Donoghue, Cyril Barinka, Jan Dvorak

**Affiliations:** 1 Department of Zoology and Fisheries, Center of Infectious Animal Diseases, Faculty of Agrobiology, Food, and Natural Resources, Czech University of Life Sciences, Kamýcká 129, 16521 Prague 6, Czech Republic; 2 Department of Parasitology, Faculty of Science, Charles University, Viničná 7, 12844 Prague 2, Czech Republic; 3 Institute of Organic Chemistry and Biochemistry, The Czech Academy of Sciences, Flemingovo N. 2, 16610 Prague 6, Czech Republic; 4 Laboratory of Structural Biology, Institute of Biotechnology, Czech Academy of Sciences, BIOCEV, Průmyslová 595, 252 42 Vestec, Czech Republic; 5 Center for Discovery and Innovation in Parasitic Diseases, Skaggs School of Pharmacy and Pharmaceutical Sciences, University of California, San Diego, 9500 Gilman Dr., La Jolla, CA 92093 USA; 6 Faculty of Environmental Sciences, Czech University of Life Sciences, Kamýcká 129, 16521 Prague 6, Czech Republic

**Keywords:** Platyhelminth, M28B metalloproteases, Prostate specific-membrane antigen, *Schistosoma mansoni*, *Fasciola hepatica*, RNA in situ hybridization, Immunohistochemistry, Folate hydrolase, NAALADase

## Abstract

**Background:**

Glutamate carboxypeptidase 2 (GCP2) belongs to the M28B metalloprotease subfamily encompassing a variety of zinc-dependent exopeptidases that can be found in many eukaryotes, including unicellular organisms. Limited information exists on the physiological functions of GCP2 orthologs in mammalian tissues outside of the brain and intestine, and such data are completely absent for non-mammalian species. Here, we investigate GCP2 orthologs found in trematodes, not only as putative instrumental molecules for defining their basal function(s) but also as drug targets.

**Methods:**

Identified genes encoding M28B proteases *Schistosoma mansoni* and *Fasciola hepatica* genomes were analyzed and annotated. Homology modeling was used to create three-dimensional models of SmM28B and FhM28B proteins using published X-ray structures as the template. For *S. mansoni*, RT-qPCR was used to evaluate gene expression profiles, and, by RNAi, we exploited the possible impact of knockdown on the viability of worms. Enzymes from both parasite species were cloned for recombinant expression. Polyclonal antibodies raised against purified recombinant enzymes and RNA probes were used for localization studies in both parasite species.

**Results:**

Single genes encoding M28B metalloproteases were identified in the genomes of *S. mansoni* and *F. hepatica*. Homology models revealed the conserved three-dimensional fold as well as the organization of the di-zinc active site. Putative peptidase activities of purified recombinant proteins were assayed using peptidic libraries, yet no specific substrate was identified, pointing towards the likely stringent substrate specificity of the enzymes. The orthologs were found to be localized in reproductive, digestive, nervous, and sensory organs as well as parenchymal cells. Knockdown of gene expression by RNAi silencing revealed that the genes studied were non-essential for trematode survival under laboratory conditions, reflecting similar findings for GCP2 KO mice.

**Conclusions:**

Our study offers the first insight to our knowledge into M28B protease orthologs found in trematodes. Conservation of their three-dimensional structure, as well as tissue expression pattern, suggests that trematode GCP2 orthologs may have functions similar to their mammalian counterparts and can thus serve as valuable models for future studies aimed at clarifying the physiological role(s) of GCP2 and related subfamily proteases.

**Graphical Abstract:**

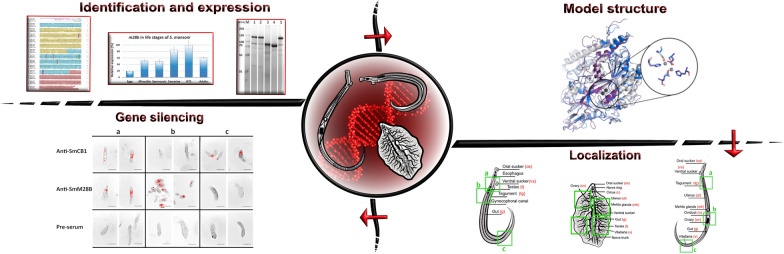

**Supplementary Information:**

The online version contains supplementary material available at 10.1186/s13071-022-05556-5.

## Introduction

Parasitic organisms affect millions of people and animals worldwide, causing serious disease. Flukes are important parasites from the class Trematoda (Platyhelminthes), and we selected two medically and veterinary important species, *Schistosoma mansoni* and *Fasciola hepatica*, as models to demonstrate putative roles of their metalloproteases from the subfamily M28B.

*Schistosoma mansoni* is a blood fluke and together with several other related species causes schistosomiasis (schistosomosis, bilharziasis) [1]. This is a chronic and potentially deadly disease with more than 200 million people infected globally [2, 3]. Their free-swimming larvae (cercariae), released into water from snail intermediate hosts, penetrate human skin and consequently migrate into the circulatory system where they mature into adult worms. Adult worms reside in the portal and mesenteric or bladder veins as male/female pairs producing hundreds of fertilized eggs per day. Chronic infection persists for years or even decades, and severe morbidity results from host immune responses to eggs in host tissues [4, 5]. Existing treatments rely heavily on one drug, praziquantel (PZQ), and no vaccine has yet been developed [2–4]. While schistosomes survive for decades in the veins of their mammalian hosts, their ability to survive and modulate host physiological processes, using a variety of secreted molecules, is crucial for survival [6]; thus, they are natural targets for specific drug/vaccine research development.

The liver fluke *F. hepatica* is among several related species and is the causative agent of fascioliasis (fasciolosis). Contrary to schistosomes, *F. hepatica* is a hermaphrodite, so it possesses both male and female reproductive organs. The disease affects livestock primarily as well as many other mammals worldwide, including humans. Cercariae are released from the intermediate snail host and encyst on the surface of herbage. Disease is transmitted by the ingestion of these cysts. After ingestion, the parasite migrates via the intestine into the peritoneal cavity and later enters the liver parenchyma, where it causes extensive tissue damage. The post-feeding larvae move to the bile duct, where they mature and produce eggs. Liver flukes generally cause serious economic losses in livestock production worldwide [7, 8], and the drug of choice is triclabendazole (TCBZ), although resistance is spreading [9]. Disease symptoms include anemia at the early stage, bile duct inflammation and fibrosis, a decrease in bile production, liver atrophy, cirrhosis, and weight loss; for some animals, disease is lethal [10]. Human infection can occur in areas where farm management is poor; thus, fascioliasis is recognized as a food-borne disease with millions of people infected or at risk [7].

In our study, we investigated two fluke species with already well-characterized genomes and body organization [11, 12] as research models to uncover and compare the physiological roles of metalloenzymes from the subfamily M28B (human GCP2 orthologs). The M28B protease family belongs to a group of membrane-bound di-zinc metalloproteases with either aminopeptidase or carboxypeptidase activity [13]. This subfamily contains proteases that are ubiquitously expressed across all phyla, from yeasts through plants to animals [14]. Additionally, homologous receptors with distinctive non-enzymatic functions, such as the transferrin receptor, also belong to the same family [14]. Human glutamate carboxypeptidase 2 (HsGCP2), also known as prostate-specific membrane antigen (PSMA), N-acetylated-alpha-linked acidic dipeptidase (NAALADase), and folate hydrolase (FOLH), is the most studied member of M28B subfamily proteases [14, 15]. In addition to GCP2, the human genome contains four other genes encoding M28B paralogs with only partially known or unknown functions [13, 16, 17]. Under physiological conditions, the primary sites of human GCP2 expression in humans include the nervous system (astrocytes and Schwann cells), kidney, prostate, salivary glands, and jejunal brush membranes [18, 19]. Despite GCP2 expression in several major human organs, there is a marked absence of data on GCP2 physiology in virtually all tissues, with the exception of the nervous system and small intestine. In the nervous system, GCP2 is involved in communication between neurons and glial cells by hydrolyzing N-acetyl-aspartyl-glutamate (NAAG), the most abundant neuropeptide in the mammalian brain. In the digestive system, GCP2 plays an important role in the absorption of dietary folates by removing their C-terminal polyglutamylated tails, thus enabling transcytosis of the resulting monoglutamylated folate [20, 21]. In addition to functioning as hydrolase/proteases, several reports allude to a non-proteolytic role(s) of GCP2 [22]. The closest human M28B paralogs to GCP2 are poorly characterized, PSMA-L expressed intracellularly [23] and GCP3 [24, 25], with 98% and 67% sequence identities, respectively. Human NAALADase L and NAALADase L2, also GCP2 orthologs, are much less characterized enzymes with likely intestine-restricted expression and aminopeptidase activity involved in protein/peptide degradation and absorption in the digestive system [15].

HsGCP2 is also a valid target for the in vivo imaging of prostate cancer because dysplastic and neoplastic transformation of prostate tissue is accompanied by a substantial increase in GCP2 expression [26]. Furthermore, inhibition of the enzyme was shown to be neuroprotective in preclinical models of numerous neurological conditions, including traumatic brain injury, neuropathic and inflammatory pain, cancer, amyotrophic lateral sclerosis [27, 28], stroke [29], and schizophrenia [30]. However, studying specific functions of GCP2 in higher organisms such as mammals is complicated because of several factors, the most notable being the presence of several paralogs; more basal models might thus be beneficial to clarify these issues.

In contrast to higher organisms, platyhelminths possess only a single gene encoding the human GCP2 ortholog [11], allowing us to study ancestral proteases before later diversification. In this study, we precisely annotated *S. mansoni* and *F. hepatica* M28B metalloproteases at the gene level. Homology modeling of both enzymes confirmed a conserved three-dimensional fold with the di-zinc active site typical for this enzyme group. Open reading frames (ORFs) for both genes were verified by sequencing, and codon-optimized versions were used for heterologous recombinant protein expressions. Despite recombinant proteins being tested for proteolytic activities in a wide range of combinatorial peptidic libraries, we did not identify any specific substrates, suggesting most likely stringent substrate specificity of the enzymes. Polyclonal antibodies and mRNA probes of both targets were produced and used for RNA in situ hybridization and immunohistochemistry to identify respective localization within parasites. For both parasites, we localized their GCP2 orthologs to reproductive, digestive, nervous, and sensory organs as well as parenchymal cells. Those localizations correlated with the expression pattern of their mammalian orthologs; thus, functional commonalities within the entire group cannot be ruled out. A comparable finding known for GCP2 KO mice [24, 25] was recorded in the case of the expression of *S. mansoni* ortholog as RNAi silencing did not lead to phenotypic changes under experimental conditions in vitro. RNAi silencing thus revealed that the genes were non-essential for immediate trematode survival under laboratory conditions. Based on our results, the real functions of trematode GCP2 orthologs remain elusive, but there are strong indications that they share common biological roles due to localizations in similar organ structures.

## Methods

### Parasite material

*Schistosoma mansoni* (a Puerto Rican strain) is routinely maintained in the laboratory in the intermediate snail host (*Biomphalaria glabrata*) and definitive host NMRI (Naval Medical Research Institute) mouse as described previously [31, 32]. Laboratory mice are maintained by a certified person (certificate number CZ 02627) in the laboratories accredited according to the animal welfare laws of the Czech Republic and EU. Free-swimming larvae (cercariae) were shed by light stimulation from infected snails into the water. Adult female mice were infected by immersing their feet and tails into 50 ml water containing approximately 200 cercariae for 45 min. Seven weeks post-infection, mice were killed by intraperitoneal injection of ketamine (Narkamon 5%—1.2 ml/kg body weight) and xylazine (Rometar 2%—0.6 ml/kg body weight) and with heparin to prevent blood clotting. Adult worms were recovered from the mouse hepatic portal system by transcardial perfusion with RPMI 160 medium. Worms in pairs were then transferred into a petri dish containing medium and chilled on ice for male and female separation by gentle prodding with a small brush. Migratory larvae (schistosomula) were prepared from cercariae in vitro and cultivated in Complete Medium 169 according to standard protocols published previously [33–35].

Living *F. hepatica* worms were collected from the liver of infected cattle (Cesky Krumlov district, Czechia) and carefully washed in phosphate-buffered saline (PBS). Worm samples were immediately frozen on dry ice and stored at − 80 °C.

### RNA isolation

Total RNA from *S. mansoni* samples (excised snail hepatopancreases with daughter sporocysts, cercariae, 5-day cultivated in vitro transformed schistosomula, adults, eggs, and miracidia) and from *F. hepatica* adults were isolated according to the TRIzol reagent (Thermo Fisher Scientific) protocol. The final precipitated RNA was air-dried and resuspended in DEPC-treated H_2_O. Single-stranded cDNA was synthesized from total RNA using SuperScript IV reverse transcriptase (Thermo Fisher Scientific) and an oligo d(T)18 reverse primer according to the manufacturer’s protocol. The resulting cDNA was purified using a QIAquick PCR purification kit (Qiagen) and stored in DEPC-H_2_O at − 20 °C.

### Sample preparation for microscopy

For RNA in situ hybridization and immunohistochemistry, the adults and schistosomula of *S. mansoni* were freshly collected and fixed in hot 4% formaldehyde heated to approximately 60 °C, but the solution did not boil (90 min for adults/30 min for schistosomula) and was cooled to 25 °C. All fixed samples were dehydrated through progressively concentrated ethanol (25%, 50%, 70%, 90%, 96%, 100% v/v ethanol) for 5 min for each step. A drop of Chromotrope 2R dye (Sigma-Aldrich) was added to the 90% ethanol step. Before embedding in paraffin, *S. mansoni* adults and schistosomula were incubated in methyl benzoate (Sigma-Aldrich) for 45 min in the case of adults and 20 min for schistosomula. Subsequently, the worms were washed twice for 5 min in benzene (Sigma-Aldrich). *Fasciola hepatica* adults were fixed in Bouin’s solution (Sigma-Aldrich) for 24 h at RT and then rinsed in PBS containing 0.02% sodium azide until the picric acid was washed off. Fixed samples were dehydrated with increasing concentrations of ethanol as was described for *S. mansoni* for 30 min for each step.

All tissues were finally embedded in paraffin-Paraplast (Sigma-Aldrich). Sections (*S. mansoni* 5 µm, *F. hepatica* 7 µm) were cut using a Shandon Finesse^®^ ME + microtome (Thermo Fisher Scientific) and placed onto X-tra adhesive slides (Leica).

### Annotation of M28B metalloproteases

Single genes coding M28B metalloproteases were identified in *S. mansoni* and *F. hepatica* based on the sequence similarities from previous sequence submissions: SmM28B as *S. mansoni* NAALADase L (GenBank XP_018651911) and FhM28B as *F. hepatica* NAALADase 2 (GenBank THD24162.1). Respective ORFs were amplified, cloned, and verified by sequencing. Fully annotated sequences were deposited into the GenBank as MZ456528 and MZ456529 for SmM28B and FhM28B, respectively.

### Homology modeling

The amino acid sequences of SmM28B and FhM28B were used for homology modeling. The 3BXM HsGCP2 structure was selected as a modeling template [36], and Modeler 9.23 software was used to construct the target-template sequence alignment and to generate a set of 3D homology models; the best model for each enzyme was selected based on discrete optimized protein energy (DOPE) scores [37]. DOPE scores for SmM28B and FhM28B homology models were − 82,422 and − 81,303, respectively. The GA341 score of both models was 1. Finally, the SmM28B and FhM28B homology models and HsGCP2 structures were superimposed in PyMol and analyzed by visual inspection.

### Cloning and mutagenesis

Codon-optimized genes encoding the SmM28B and FhM28B proteins were custom-made by the Thermo Fisher gene-string synthesis protocol. Coding sequences were PCR amplified with corresponding sets of gene-specific primers (Additional file 1: Table S1), and pD221 donor vectors were constructed using the BP Gateway cloning protocol (Invitrogen). Expression plasmids featuring N-terminal purification His-Strep-HALO tags were prepared by recombining the donor vectors and the in-house expression pDEST320 destination vector (Additional file 2: Fig. S1) using the LR Gateway reaction mix. SmM28B(E439M) and FhM28B(E413M) mutants, harboring inactivating mutations of the putative proton shuttle glutamate, were constructed by Quick-change site-directed mutagenesis using corresponding expression plasmids as templates. To this end, target glutamate residues were mutated to methionine via PCR with mutagenic primers (Additional file 1: Table S1) followed by elimination of the template by DpnI treatment, as described for the HsGCP2 ortholog [38]. *Escherichia coli* clones transformed by a plasmid containing the modified sequence were verified using Sanger sequencing.

### Expression and purification of trematode M28B metalloproteases in HEK-293T cells

For eukaryotic expression, metalloproteases were cloned into the pDEST320 destination vector in frame with the TEV-cleavable His-Strep-HALO tag (Additional file 2: Fig. S1) and expressed and purified essentially as described previously [39]. Briefly, all variants were expressed using HEK-293T/17 cells following transient transfection, mediated by linear polyethyleneimine (PEI) (Polysciences). The suspension culture was grown in Erlenmeyer flasks in FreeStyle F17 medium (Thermo Fisher Scientific) at 110 rpm under a humidified 5% CO_2_ atmosphere at 37 °C. For large-scale expression, an expression plasmid was diluted in PBS (1 mg in 25 ml of PBS for 1 l of the cell culture). Then 3 ml of the PEI stock solution (1 mg/ml) was added; the mixture was vortexed briefly, incubated for 10 min at room temperature, and then added to the cell suspension (4 × 10^6^ cell/ml). Four hours post-transfection, the cell suspension was diluted with an equal volume of ExCell serum-free medium. Cells were harvested 72 h post-transfection by centrifugation at 500 g for 5 min, and then the cell pellet was frozen in liquid nitrogen and stored at − 80 °C until further use.

The cell pellet was lysed by sonication in ice-cold lysis buffer (100 mM Tris–HCl, 10 mM NaCl, 5 mM KCl, 2 mM MgCl_2_, 10% glycerol, 0.2% Nonidet P-40, pH 8.0) supplemented with the protease inhibitor cocktail cOmplete EDTA-free (Roche). Following incubation on ice for 30 min, the cell lysate was cleared by centrifugation at 40,000 *g* for 30 min, and the supernatant was loaded on a Strep-Tactin column (IBA-Lifesciences). The fusion was eluted with the lysis buffer supplemented with 2 mM desthiobiotin. Alternatively, when deemed beneficial, the N-terminal fusion tag was cleaved-off by TEV protease (at a 1:10 TEV to target ratio) at 8 °C overnight. The final purification step comprised size exclusion chromatography on a Superose 6 column (GE Life Sciences) with 20 mM MOPS, 150 mM NaCl, pH 7.4, as a mobile phase. Purified proteins were concentrated at 1 mg/ml and flash-frozen in liquid nitrogen until further use.

### Recombinant production of SmM28B in *E. coli*

For polyclonal antibody production, the SmM28B antigen was heterologously expressed in a prokaryotic expression system. The codon-optimized sequence encoding SmM28B was cloned into the pEC527 destination vector (a gift from Dominic Esposito; Addgene plasmid #11518) in frame with the TEV-cleavable hexahistidine tag. The recombinant protein was expressed in *E. coli* BL21(DE3) cells in LB medium at 37 °C upon 1 mM IPTG induction and further purified from inclusion bodies using Ni^2+^ chelating chromatography under denaturing conditions. Briefly, the cell pellet was disrupted by sonication in PBS, and inclusion bodies (IBs) were isolated by centrifugation at 5000 *g* for 10 min. IBs were further purified by consecutive sonication-assisted solubilization in PBS + 1 M urea, PBS + 1 M NaCl, and PBS + 1% Triton X-100, where each solubilization step was followed by centrifugation at 5000 *g* for 10 min. Following the final centrifugation, the IBs were dissolved in an equilibration buffer (10 M urea, 100 mM Tris–HCl, pH 8.0) and centrifuged at 30,000 *g* for 30 min, and the supernatant was loaded onto a 5-ml Ni–NTA column. His-tagged SmM28B was eluted with the equilibration buffer supplemented with 200 mM imidazole. Fractions containing the purified protein were pooled and used to prepare polyclonal rabbit antiserum.

### Expression and purification of human GCP2

Expression and purification of the extracellular part of HsGCP2 (residues 44-750) were carried out as described previously [40].

### Aminopeptidase library screening

The aminopeptidase activities of GCP2-like enzymes were evaluated using the library of proteinogenic amino acids, except cysteine, labeled with a fluorophore, 7-amino-4-methylcoumarin (AMC). The enzymatic assays were carried out in 384-well black plates in a final volume of 50 µl. The reaction mixtures consisted of 5 µM of individual fluorophore-labeled amino acids and 500 nM of enzyme in an assay buffer comprising 50 mM Tris–HCl, 150 mM NaCl, 0.001% C_12_E_8_ (dodecyloctaglycol), pH 7.4. The reactions were incubated at room temperature for 60 min, and the release of free AMC was monitored using a CLARIOstar fluorimeter (BMG Labtech GmbH) with excitation/emission wavelengths set at 365 and 440 nm, respectively. The two-fold dilution series of AMC with a starting concentration of 5 µM was used as a positive control and for quantification of the reaction products. The substrate without enzyme was used as a negative control and as a signal background.

### Carboxypeptidase library screening

The carboxypeptidase activities of GCP2 enzymes were evaluated using the library of dipeptides. The library was synthesized as 19 mixtures, each containing a defined N-terminal residue and a mixture of 19 proteinogenic amino acids at the C-terminus. Cysteine was not present in the library. Enzymatic assays were carried out in 96-well plates with a final volume of 50 µl. Reaction mixtures consisted of 500 µM peptide mixture and 500 nM enzyme in the assay buffer. Reaction mixtures were incubated at room temperature for 60 min, quenched by the addition of 0.1% trifluoroacetic acid in 5% acetonitrile, and released amino acids were quantified by Waters Corporation AccQ-Tag Ultra chemistry package on RP-HPLC (Shimadzu, HPLC Prominence system) according to the manufacturer's instructions. The complementary peptide library comprising a defined C-terminal residue and a mixture of 19 proteinogenic amino acids at the N-terminus was used for the control experiments.

### Multiplex substrate profiling by mass spectrometry (MSP-MS)

The MSP-MS assays were performed as previously described [31]. Briefly, protease samples were incubated in triplicate reaction tubes with a mixture of 228 synthetic tetradecapeptides (0.5 μM each) in the reaction buffer. Then, 20 μl of the reaction mixture was removed from each replicate after a set incubation time (15, 60, 120, 240, and 1200 min of incubation), and enzyme activity was quenched by adding 40 μl of 6.4 M guanidinium chloride (GuHCl). Samples were flash frozen immediately at − 80 °C. A control reaction consisted of enzyme pre-treated with GuHCl prior to peptide library exposure. All samples were subsequently thawed, acidified to pH < 3.0 with 1% formic acid, desalted with C18 LTS tips (Rainin), injected into a Q Exactive Mass Spectrometer (Thermo) equipped with an Ultimate 3000 HPLC (Thermo), and analyzed as previously described [32].

### RT-qPCR analysis of *S. mansoni* gene expression

RT-qPCR involved specific primers designed and selected for gene targets encoding SmM28B, *S. mansoni* cathepsin B1.1 (SmCB1.1, GenBank AJ506157), and *S. mansoni* cytochrome C oxidase I (SmCOX I, GenBank AF216698) as the sample reference gene transcript (Additional file 3: Table S2). Primer3 software (http://frodo.wi.mit.edu/ [41]) was used to design specific novel primers for the gene encoding SmM28B. Primers were evaluated by serial dilutions of both the primers and the cDNA template as described [33, 42, 43], while SmCB1.1 primers were adopted from our previous studies [33, 43]. The cDNA from various life stages was generated using the mRNA isolation protocol described above and previously [33, 43]. Reactions containing LightCycler 480 SYBR Green I Master (Roche) were prepared in final volumes of 25 μl in 96-well plates and carried out as described previously [33]. PCR reactions were performed in triplicate with at least one biological replicate. Analysis using the reference gene transcript and the 2^−CT^ method was as previously published [33, 43] to measure transcript levels [44]. The whole experiment was repeated when CT values of technical replicates fluctuated by 0.5 or more. The resulting transcript levels were calculated as a percentage of the nonspecific control (mCherry dsRNA for RNAi) or the highest transcript level that was set at 100%.

### Probes design for RNA in situ hybridization

The specific PCR products were amplified by polymerase chain reaction (PCR) from the first-strand cDNA of adults *S. mansoni* and *F. hepatica* using gene-specific primers (Additional file 4: Table S3). The PCR fragments of approximately 450 bp were ligated into the pGEM-T Easy Vector (Promega), and cloned sequences were verified by DNA sequencing. Verified constructs were linearized and used as a template for the production of sense/antisense RNA probes according to the manufacturer’s instructions (DIG RNA labeling kit SP6/T7, Roche).

### RNA in situ hybridization (ISH)

Sections (7 µm *F. hepatica*/5 µm *S. mansoni*) of adult worms were de-paraffinized in xylene, rehydrated, and washed in diethyl-pyrocarbonate (DEPC, Sigma-Aldrich)-treated water. The *S. mansoni* sections were incubated in 0.01 M sodium citrate buffer, pH 6.0, in a boiling water bath for 15 min and were then cooled for 30 min. In the case of *F. hepatica*, the sections were treated with proteinase K (final concentration 2 µg/ml, Roche) for 5 min at 37 °C. Hybridization was performed for 16 h at 42 °C (*S. mansoni*) and 55 °C (*F. hepatica*) with RNA probes diluted to 1:200 in a hybridization mixture (5 × SSC, 1 × PBS, 0.1% Torula yeast RNA (Sigma-Aldrich), 50% formamide, 10% dextran sulfate molecular weight 4000 (Sigma-Aldrich), 1% Tween 20) according to the modified protocols [45, 46]. After hybridization, slides were washed and incubated in blocking solution as described [45, 46]. Subsequently, final detection in the tissue of *F. hepatica* was carried out using Anti-DIG-AP antibodies (Roche) at a 1:500 dilution and SIGMAFAST™ Fast Red TR/Naphthol AS-MX tablets (Sigma-Aldrich); results were visualized with a Nikon ECLIPSE Ni-E microscope. In the case of the significantly smaller *S. mansoni*, hybridized probes were labeled with Anti-DIG-HRP antibodies (Roche) at a 1:500 dilution, and signal amplification was performed using the Tyramide Signal Amplification (TSA) system with the Cyanine Plus 5 Tyramide Reagent fluorescence system (Perkin Elmer) according to the manufacturer’s protocol. Sections were embedded in ProLong™ Diamond Antifade Mountant (Thermo Fisher Scientific) and visualized with an Olympus IX83 microscope equipped with PCO edge 5.5. camera and CoolLED pE-4000 LED illumination system. As negative controls, sections were incubated under the same conditions with sense probes and without any probe. Images from both *S. mansoni* and *F. hepatica* slides were processed using ImageJ software version 1.52 u [47].

### Polyclonal antibody production and immunohistochemistry (IHC)

Purified recombinant SmM28B (BL21 *E. coli*) and FhM28B (HEK-293FT source, with the N-terminal HALO tag removed) were used to produce rabbit polyclonal antibodies at the service facility of the Institute of Microbiology, Czech Academy of Sciences. Rabbit polyclonal antibodies against major *S. mansoni* gut protease SmCB1 were raised against recombinantly produced protein [48] and kindly provided by Dr. Daniel Sojka, from the Biology Centre, Czech Academy of Sciences. Sample fixation, deparaffinization, rehydration, and antigen retrieval followed the same protocol as was described in the RNA in situ hybridization section below [49, 50]. After antigen retrieval with sodium citrate buffer, pH 6.0, sections were washed three times in 1 × PBS. Then, sections were permeabilized in fresh PBS, pH 7.5, supplemented with 0.25% (v/v) Triton X-100 (PBS-Tx) for 20 min followed by overnight incubation in the blocking buffer composed of PBS-Tx + 2% BSA. Finally, the sections were washed once in antibody diluent (AbD; PBS-Tx + 1% BSA) and subsequently probed with respective immunized sera at a dilution of 1:50. Slides were incubated in the wet chamber at 4 °C overnight. Respective controls included primary sera and no sera to exclude non-specific reactions of sera or secondary antibodies, respectively. After three washes in (PBS-Tx) for 10 min each, slides were incubated with goat anti-rabbit IgG H&L Alexa Fluor Plus 647 secondary antibodies (ThermoScientific) at a dilution of 1:500, followed by 45 min of incubation at 37 °C in the wet chamber. Slides were again washed 3 × in PBS-Tx for 10 min each followed by a final 10 min wash in PBS. Slides were mounted in ProLong Diamond Antifade Mountant with DAPI (Thermo Fisher Scientific) and visualized by Nikon ECLIPSE Ni-E fluorescence microscope. Images were processed with ImageJ software version 1.52u.

### RNA interference in *S. mansoni*

As templates for dsRNA synthesis, PCR products of targeted SmM28B and positive control *S. mansoni* cathepsin B1.1 (SmCB1.1, GenBank AJ506157) were PCR amplified from cDNA prepared from 5-day-old in vitro-cultivated *S. mansoni* schistosomula as described previously [33]. DNA coding mCherry protein was used as a nonspecific control dsRNA. PCR products were approximately 500 bp in size (Additional file 5: Table S4). Synthesis of dsRNA from gel-purified PCR templates was accomplished using the T7 RiboMAX Express RNAi System (Promega) as described previously [33]. Schistosomula transformed from cercariae or adults perfused from the infected mice [33–35] were incubated in 24-well plates containing 1 ml of Complete Medium 169 with 5% FBS [34], and dsRNA 30 µg/ml at 37 °C and 5% CO_2_ [33]. (i) For monitoring of gene silencing levels, parasites were collected after 5 days of cultivation, washed three times in PBS, and resuspended in 50 ml of Trizol reagent (Thermo Fisher Scientific). RNA isolations and evaluations of gene expression levels by RT-qPCR followed previous protocols [33, 43] as described for the reaction design above. (ii) For observing phenotypic changes, dsRNA-treated parasites were monitored regularly for 14 days.

## Results

### Identification of M28B metalloproteases in *S. mansoni* and *F. hepatica*

**Fig. 1:**
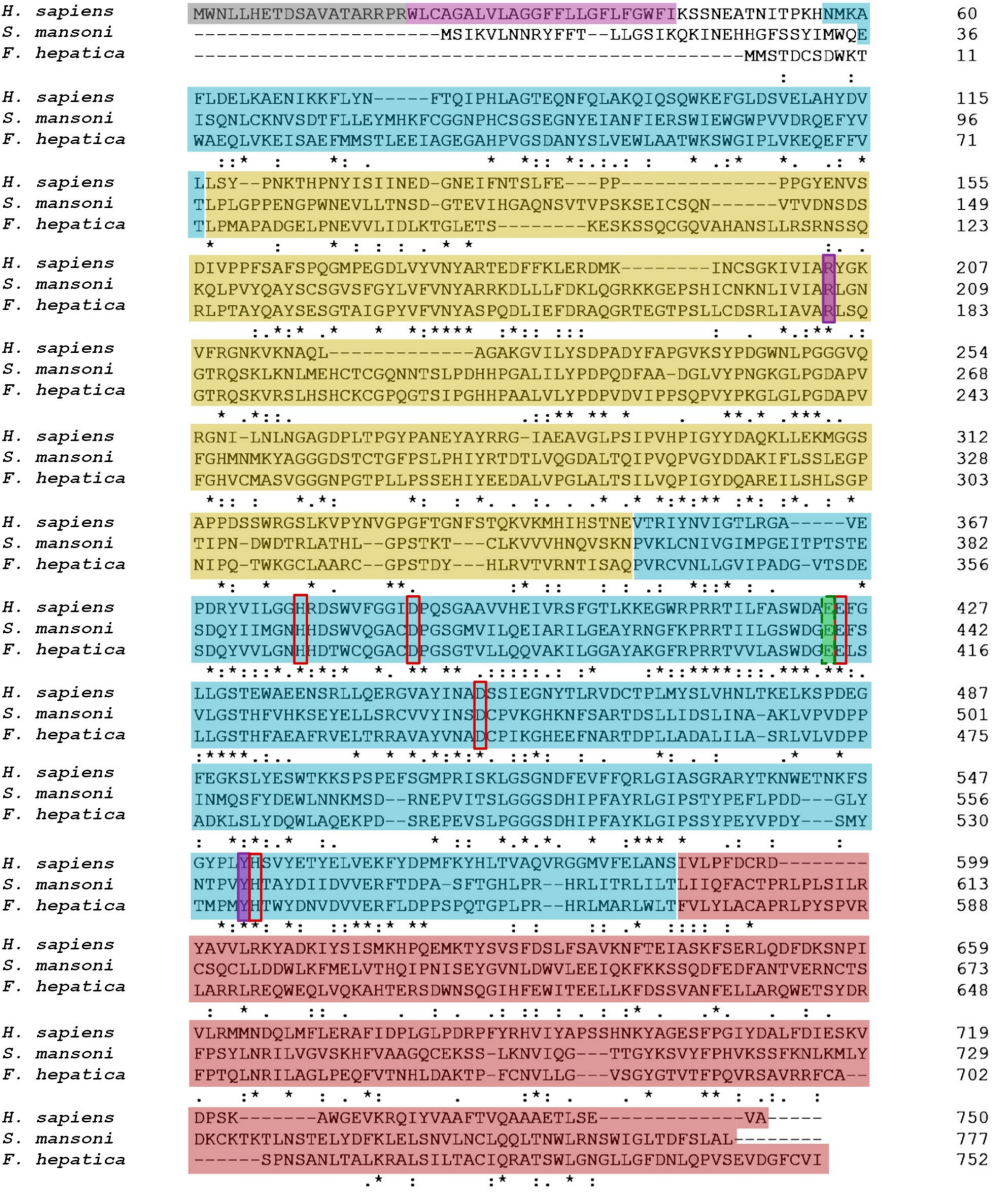
Sequence alignment of SmM28B, FhM28B, and HsGCP2. The intracellular part and the transmembrane domain of human GCP2 are shaded *gray* and *magenta*, respectively. Protease-like (*blue*), apical (*yellow*), and C-terminal dimerization (*red*) domains are present. The catalytic acid/base glutamate is highlighted by the green background in the rectangular box. Residues coordinating zinc ions are marked by red rectangular boxes, and residues coordinating α-carboxylate binding are shaded in the violet background in rectangular boxes

The sequence alignment of *S. mansoni* and *F. hepatica* M28B proteases with their human GCP2 ortholog is shown in Fig. 1. The SmM28B and FhM28B protein sequences share 48% and 30% similarity with HsGCP2, respectively. All three proteins share an identical domain organization (also verified by homology modeling below) comprising the protease, apical, and C-terminal dimerization domain, which is responsible for protease dimer formation [51]. At the same time, both SmM28B and FhM28B lack the intracellular and transmembrane domains present in HsGCP2, indicating their intracellular functions or a different secretory mechanism. Importantly, the catalytic glutamate residue, as well as residues responsible for coordinating active-site zinc ions, are conserved in FhM28B and SmM28B, pointing toward their putative proteolytic functions (Fig. 1).

### Homology modeling

**Fig. 2:**
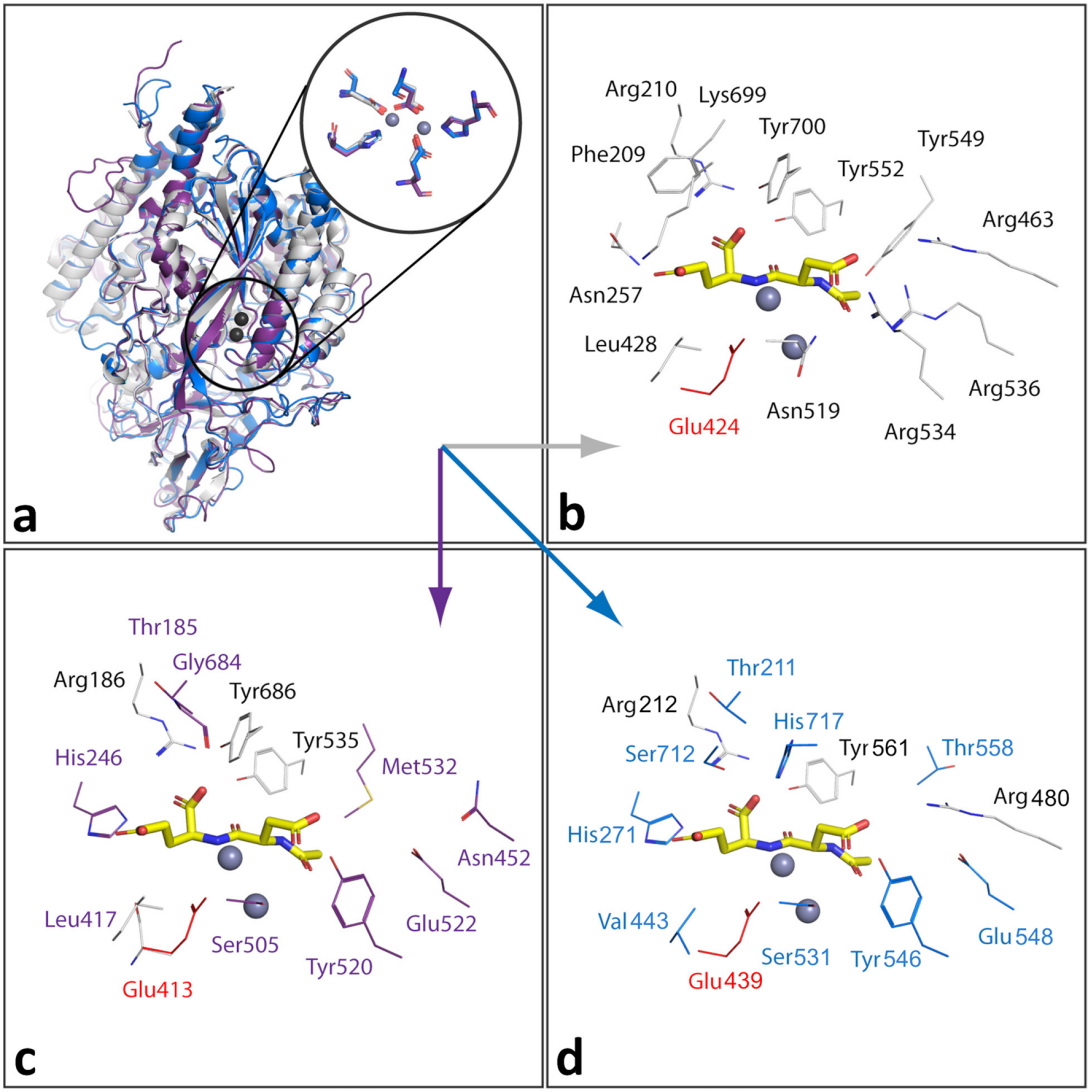
Homology models of SmM28B and FhM28B. (**a**) The superposition of the HsGCP2 structure (pdb: 3BXM, gray cartoon representation), SmM28B (blue), and FhM28B (violet) homology models reveals the conservation of their overall fold and the coordination sphere of active-site zinc ions (gray spheres). The major differences are observed in residues forming the substrate-binding pocket. (**b**) Residues delineating the substrate-binding pocket of HsGCP2 are shown as gray lines. The active site-bound NAAG is shown in yellow stick representation. The FhM28B (**c**) and SmM28B (**d**) residues that differ from the human ortholog are highlighted in violet and blue, respectively. The critical proton shuttle glutamate residue is highlighted in red and was mutated to methionine to prepare inactive variants of the enzymes. The figure was generated using PyMol 2.4.1

To provide structural insight into SmM28B and FhM28B, we constructed homology models of SmM28B and FhM28B and compared them with the X-ray crystal structure of HsGCP2 (PDB code 3BXM). As shown in Fig. 2, the overall fold of all three proteins was virtually identical. The active site of HsGCP2 comprises two zinc ions that are critical for GCP2 folding and enzymatic activity [52]. Our homology models revealed that the three-dimensional arrangement of the active sites, including residues coordinating Zn^2+^ ions of GCP2, were conserved in *S. mansoni* and *F. hepatica* orthologs. Similarly, Glu424, the proton shuttle residue in HsGCP2, which is essential for its catalytic activity, was also conserved in studied orthologs. Consequently: (i) it can be assumed that both orthologs might retain protease activity; (ii) based on these predictions we designed E413M and E439M inactive mutants for FhM28B and SmM28B, respectively, which served as negative controls for activity profiling (see below; [38]).

Analysis of the homology models further highlighted marked differences in the characteristics of residues delineating substrate specificity pockets in the internal cavity of the enzyme (Fig. 2, Refs. [53, 54]). The positively charged arginine patch, comprising residues Arg463, Arg534, and Arg536, is the hallmark of the non-prime pocket of HsGCP2 and imparts specificity for negatively charged residues at the P1 position of HsGCP2 substrates (Asp, Glu) [54]. Corresponding residues in SmM28B are Arg480, Tyr546, and Glu548, while, in FhM28B, these positions are occupied by Asn452, Tyr520, and Glu522. Similarly, Arg210 and Lys699, interacting with α- and γ-carboxylates of the C-terminal glutamate residues in HsGCP2 substrates, respectively, are critical determinants of HsGCP2 substrate specificity in the S1’ pocket [53, 55, 56]. However, while residues corresponding to Arg210 are conserved in both orthologs, Lys699 is replaced by small uncharged Ser712 and Gly684 in SmM28B and FhM28B, respectively. Overall, these findings point towards different substrate specificities of the two orthologs when compared to the HsGCP2 preference for negatively-charged glutamate-containing substrates [55].

### Heterologous expression and purification of SmM28B and FhM28B

**Fig. 3:**
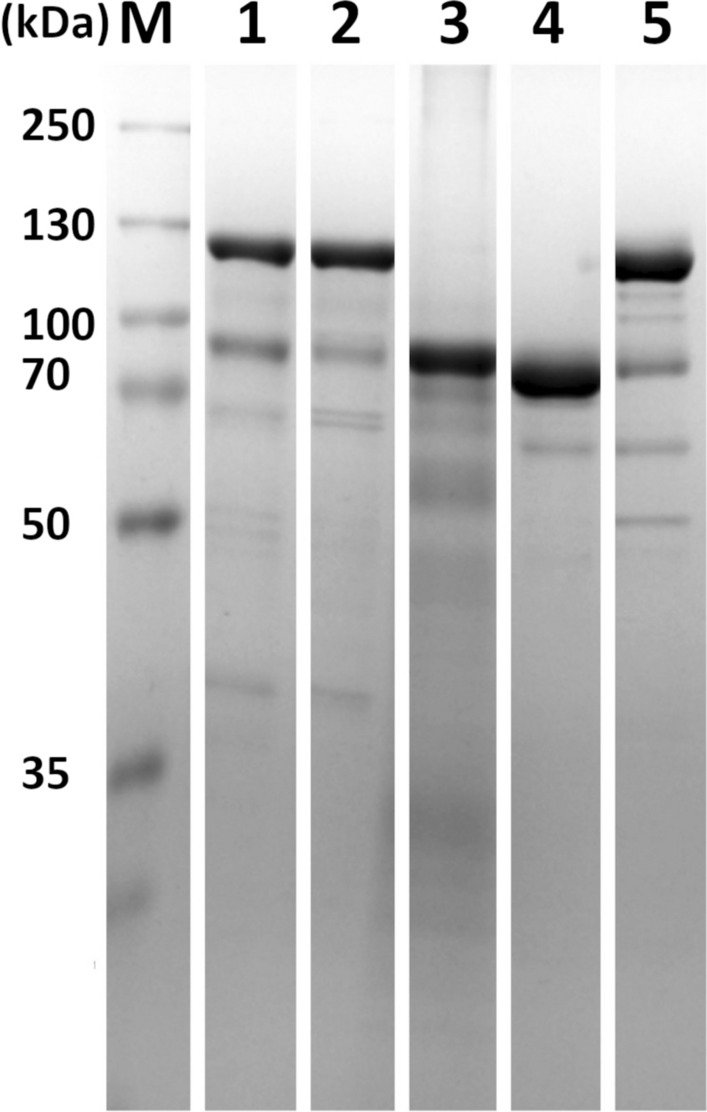
SDS-PAGE analysis of purified recombinant SmM28B and FhM28B. Lanes **1** through **5** show HALO-SmM28B (123 kDa), HALO-SmM28B (E439M) (123 kDa), His-SmM28B (85 kDa), FhM28B (83 kDa), and HALO-FhM28B (E413M) (126 kDa), respectively. Calculated molecular masses of individual constructs are shown in brackets. His-SmM28B was expressed in *E. coli*, while the remaining constructs were expressed in HEK-293FT cells as fusion proteins with the N-terminal TEV-cleavable His-Strep-HALO tag. For FhM28B, the N-terminal tag was cleaved off by TEV protease. Proteins were separated by reducing SDS-PAGE and stained by Coomassie Brilliant Blue R-250

Both orthologs were successfully cloned into vectors for heterologous expression in HEK-293T/17 that are more suitable for the production of complex, multidomain proteins. All wild-type enzymes, together with their corresponding putative inactive mutants, were purified from HEK-293T/17 lysates using Strep Tactin affinity chromatography. For the FhM28B construct, the purification protocol further followed comprised by a size-exclusion chromatography step. Finally, for antibody preparation, the HALO-FhM28B fusion was cleaved by TEV protease so only the FhM28B ortholog without the tag was used for rabbit immunization (Additional file 6: Fig. S2). The overall purity, as estimated by Coomassie-stained SDS-PAGE, was 60% and 90% for wild-type SmM28B and wild-type FhM28B, respectively (Fig. 3). Importantly, the identities of all constructs were confirmed by mass spectrometry, and elution profiles from size-exclusion chromatography revealed a predominant monodisperse peak corresponding to the expected dimeric molecular mass, indicating the production of correctly folded protein species that were further used for profiling of peptidase activities. Additionally, recombinant SmM28B was also expressed in *E. coli* and purified by Ni–NTA chromatography (Fig. 3), and this recombinant protein, together with TEV-cleaved FhM28B described above, was used for the production of polyclonal rabbit antibodies for immunohistochemistry experiments.

### Profiling peptidase activities

**Fig. 4:**
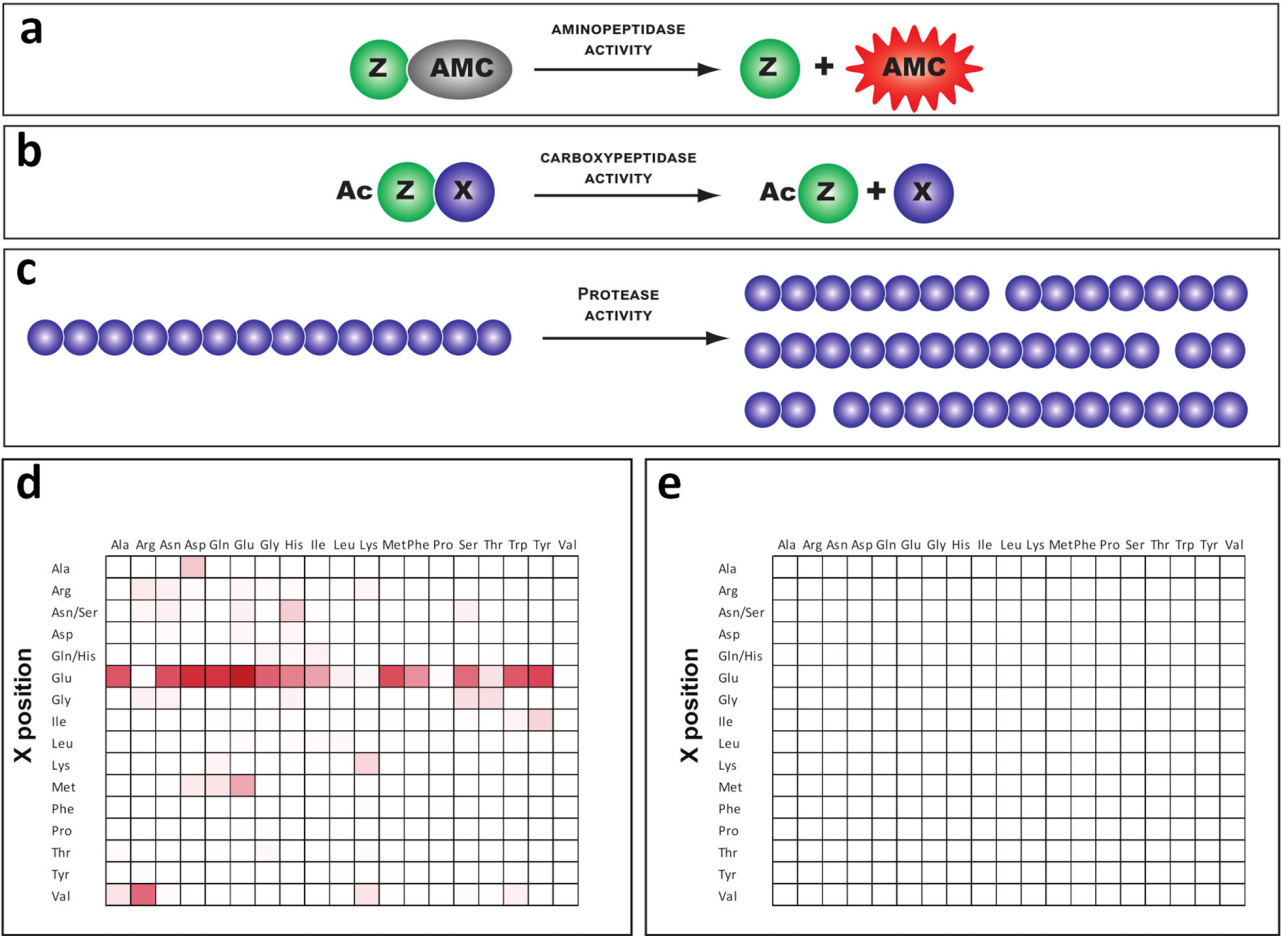
The scheme of aminopeptidase library (**a**), carboxypeptidase library (**b**), and MSP-MS library screen (**c**). The results of HsGCP2 (**d**) and FhM28B (**e**) screening against the libraries. The data are presented in the form of heat maps with 0% and maximum 60% substrate conversion colored white and red, respectively

Given the structural conservation of the active sites of HsGCP2 orthologs, we hypothesized that trematode enzymes should have intrinsic peptidase activity, although with specificity differing from human counterparts because of marked amino acid variations in the S1 and S1′ pockets. To evaluate the enzymatic activity of HALO-FhM28B, we used three complementary approaches for profiling aminopeptidase, carboxypeptidase, and endopeptidase enzymatic activities against specific combinatorial peptidic libraries (Fig. 4). HsGCP2 and the HALO-FhM28B (E413M) were used as positive and negative controls, respectively. We also included HALO-SmM28B (and HALO-SmM28B (E439M) as a negative control) in preliminary activity screens, but, upon data evaluation, we decided not to pursue these constructs further because of insufficient purity and the high likelihood of false positives stemming from the presence of contaminating cellular peptidases. The results from carboxypeptidase activity screening are shown in Fig. 4. While substrate preferences of HsGCP2 were consistent with previously published data and thus provided confirmation of the usability of our assay conditions, carboxypeptidase activity was not detected for either wild-type HALO-FhM28B or the HALO-FhM28B (E413M) mutant. Similarly, no aminopeptidase or endopeptidase activities were identified when using either traditional aminopeptidase substrates, represented by amino acids labeled by AMC at their N-terminus, or by the 228-peptide library of tetradecapeptides, respectively (data not shown).

### SmM28B expression is present in all life stages of *S. mansoni*

**Fig. 5:**
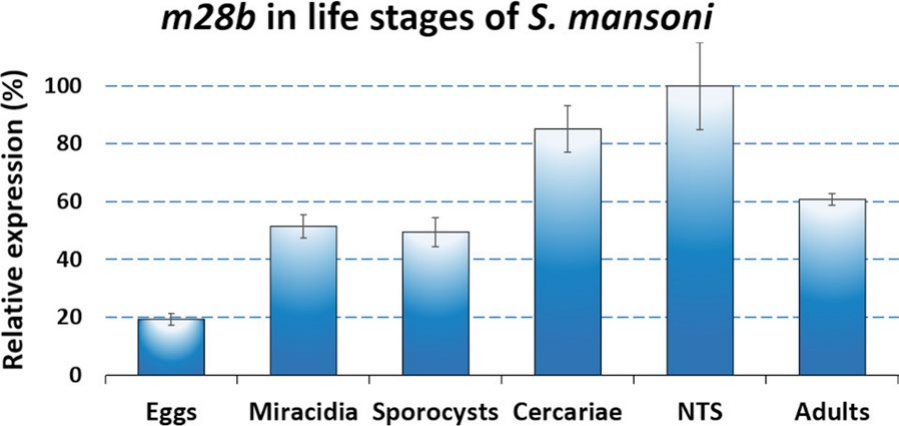
Evaluation of the expression levels of the gene encoding SmM28B by the RT-qPCR in life stages of *S. mansoni*. *Smcoxi* was used as a suitable housekeeping gene for normalization of gene expression in samples of different life stages of *S. mansoni*

Expression levels of the gene encoding SmM28B were evaluated by RT-qPCR in the life stages of *S. mansoni.* The life stages for analysis were: stages residing in the mammalian host (newly transformed schistosomula, adults, and eggs); miracidia (infecting snail host); daughter sporocysts (producing cercariae within the snail); cercariae (leaving snail host and penetrating the skin of the mammalian host). Expression was normalized to the expression of the *smcoxi* gene and was observed, constitutively, with a slight increase in more motile stages (Fig. 5).

### Localizations of M28B metalloproteases in *S. mansoni* and *F. hepatica* adults

**Fig. 6:**
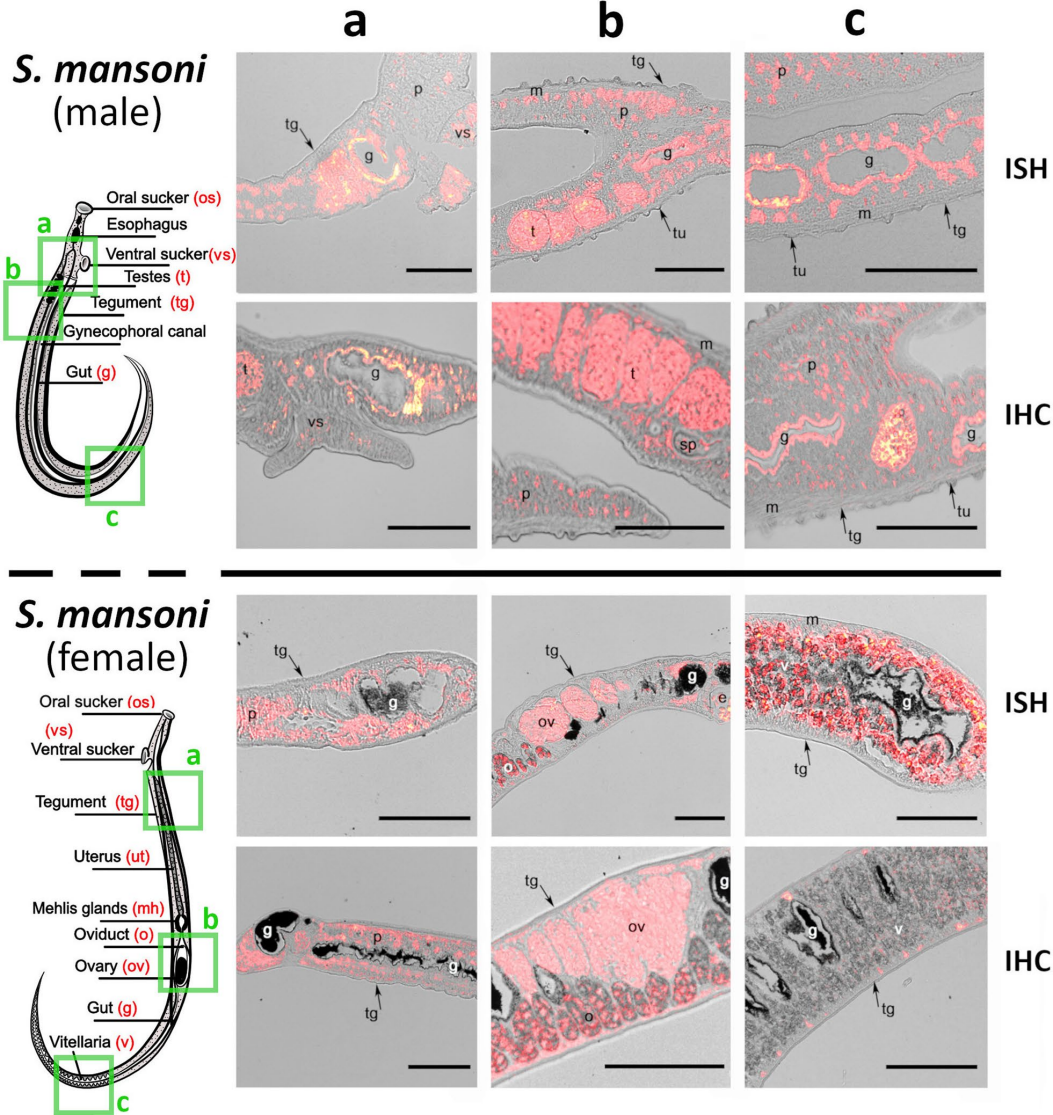
Localization of SmM28B and M28B in adult male and female of *S. mansoni*. ISH-reaction of antisense DIG-labeled RNA probes designed to label SmM28B sense mRNA (protein-coding mRNA) with histological sections (5 µm) of *S. mansoni* adult (red). IHC—reaction of polyclonal antibodies against SmM28B with sections of *S. mansoni* adult (red). Adults were monitored in three parts: (**a**) head part; (**b**) middle part; and (**c**) posterior part of the worm with the focus on parenchyma, gut, and tegument. The green squares on the left diagram indicate where the cross-sections were taken. SmM28B peptidase was detected in ovary (ov), oviduct (o), vitellaria (v), testes (t), and parenchyma (p) of both sexes. Interestingly, SmM28B was localized exclusively in the gastrodermis of the male but not in the female. SmM28B was not detected in muscles (m), tegument (tg), and tubercules (tu) of both sexes. The scale bars represent 100 µm

**Fig. 7:**
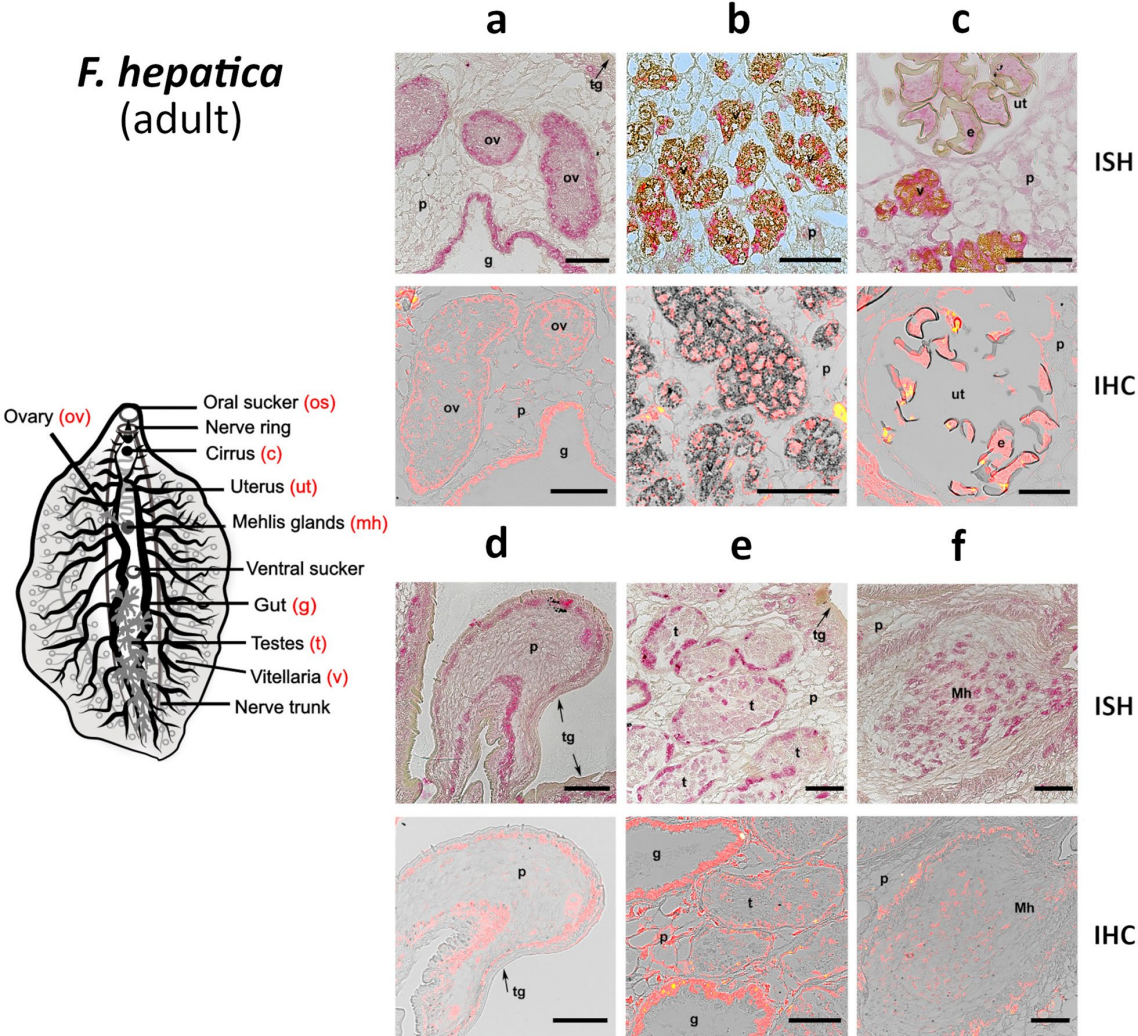
Localization of mRNA encoding FhM28B and M28B protein in *F. hepatica* adults. ISH reaction of antisense DIG-labeled RNA probes designed to label FhM28B sense mRNA (protein-coding mRNA) with histological sections (7 µm) of *F. hepatica* adult (red). IHC—a signal of polyclonal antibodies against SmM28B with sections of *F. hepatica* adult (red). Adults were monitored in six parts: (**a**) ovary; (**b**) vitellaria; (**c**) uterus; (**d**) parenchyma and tegument; (**e**) testes; (**f**) Mehlis glands. FhM28B transcripts/proteins were detected in ovary (ov), eggs (e), vitellaria (v), Mehlis glands (Mh), gut (g), and parenchyma (p). FhM28B was not detected in uterus (ut), muscles (mu) and tegument (tg). The scale bars represent 100 µm

To identify spatial gene expression of M28B proteases within the tissues of *S. mansoni* and *F. hepatica* adults, RNA in situ hybridization and indirect immunofluorescence microscopy demonstrated that enzymes occur with the same pattern in both species and follow the distribution of mammalian orthologs (Figs. 6, 7). Results showing localization of M28B on transcript and protein levels in both flukes are summarized in Table 1. Localization patterns in *S. mansoni* and *F. hepatica* (Figs. 6, 7) were identically detected in the reproductive organs and eggs of both flukes. Additionally, localization of the studied enzymes was confirmed in parenchyma and gastrodermis, except for *S. mansoni* females, where a signal in the gastrodermis was missing. In agreement with previous findings of such GCP2 orthologs, we were able to detect enzymes in the cerebral tissue of both species (Additional file 7: Fig. S3), despite not being confirmed by IHC in *F. hepatica*. Interestingly, the control sense probe revealed anti-sense transcripts (i.e. non-coding RNAs) in *S. mansoni* vitellaria and oviducts (Additional file 8: Fig. S4).

**Table 1:** Summary of M28B localization sites in model organisms *S. mansoni* and *F. hepatica*

Organ structure	*S. mansoni*				*F. hepatica*	
	Male		Female		Adult	
	ISH	IHC	ISH	IHC	ISH	IHC
Cirrus	−	−			**+**	**+**
Egg			−	−	−	**+**
Gastrodermis	**+**	**+**	−	−	**+**	**+**
Muscles	−	−	−	−	−	−
Nerve ring/cerebral ganglia	**+**	**+**	**+**	**+**	**+**	−
Ovary			**+**	**+**	**+**	**+**
Oviduct			**+**	**+**	−	−
Parenchyma	**+**	**+**	−	**+**	−	**+**
Seminal vesicles	−	**+**			−	−
Tegument	−	−	−	−	−	−
Testes	**+**	**+**			**+**	**+**
Uterus			−	−	−	−
Vitellaria			**+**	**+**	**+**	**+**

+, positive signal; −, negative signal; ISH (RNA in situ hybridization), IHC (immunolocalization)

### RNAi-mediated knockdown of SmM28B expression

**Fig. 8:**
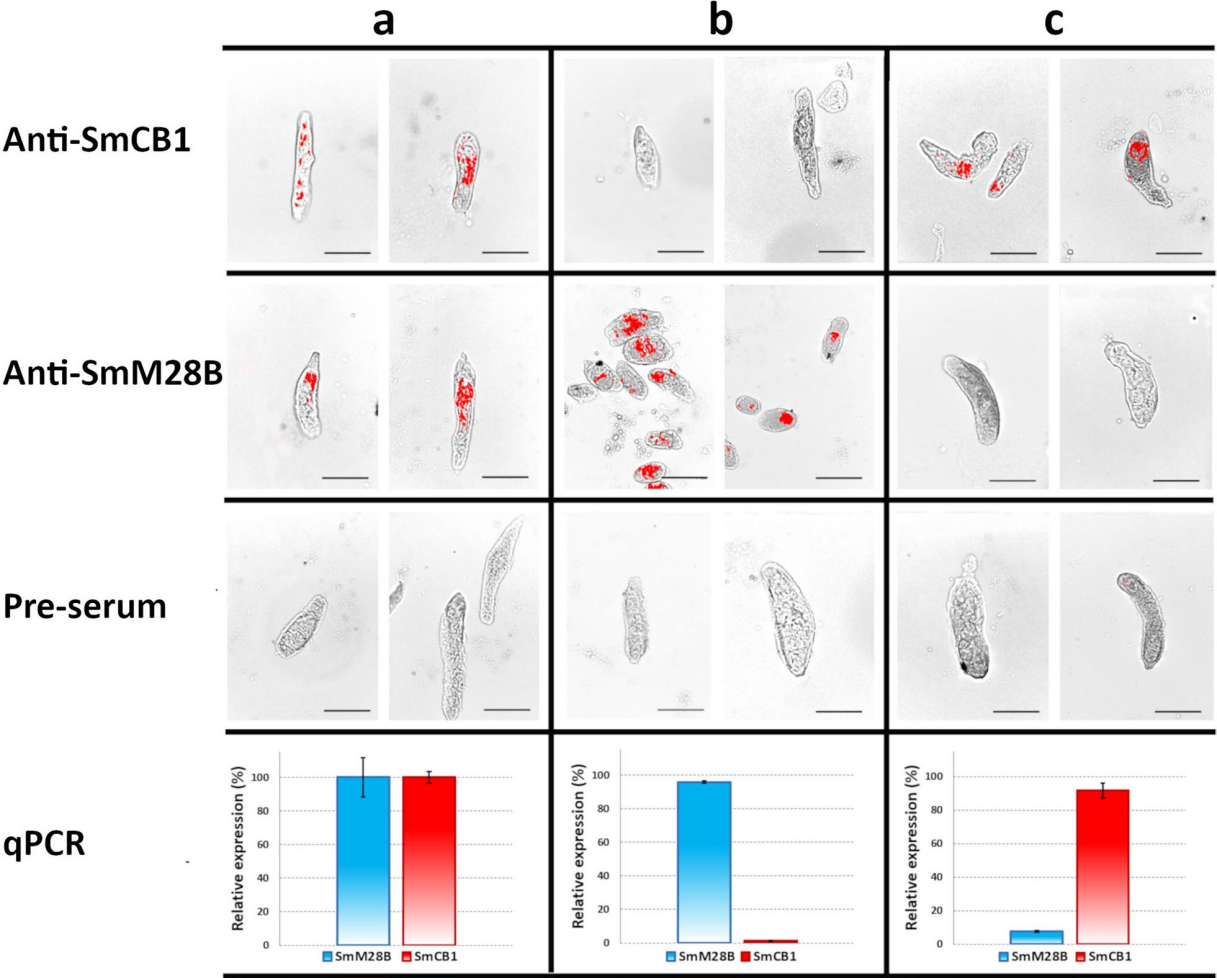
Immunolocalization on schistosomula after 5 days of exposure to RNAi treatment. The panel presents the result of immunolocalization of SmM28B on *S. mansoni* schistosomula exposed to RNAi treatment. Sections with schistosomula were probed with anti-SmM28B, anti-SmCB1, and pre-serum respectively. Column (**a**) represents the result of immunohistochemistry on schistosomula treated with mCherry dsRNA serving as a control for RNAi. Column (**b**) presents the results of schistosomula treated with SmCB1 dsRNA. Column (**c**) shows the result of schistosomula treated with M28B dsRNA. All samples were probed with antibodies against SmCB1, SM28B, and control pre-serum. The qPCR charts show levels of SmM28B and SmCB1 expression in percentages compared to the controls. The scale bar represents 50 µm

Knockdown was tested on newly transformed schistosomula and adults of *S. mansoni *in vitro. Parasites were exposed by soaking to specific dsRNA for 5 days; subsequently, levels of remaining mRNAs coding particular proteins were evaluated by RT-qPCR. RNAi reduced expression of SmM28B by around 95% in schistosomula (Fig. 8), while in the adult worm knockdown did not exceed 30% (not shown). Our positive control targeting the major gut protease SmCB1 was around 98% in schistosomula (Fig. 8), which is consistent with our previous observations [33]. Despite significant knockdown of SmM28B expression, any measurable phenotypic changes were not observed in the parasites. Successful silencing of the major gut protease SmCB1 (a positive control) and SmM28B expressions in schistosomula were also confirmed visually by immunolocalization. The fluorescent localization signals of SmM28B detected in the head area, gut, and esophageal region of schistosomula and SmCB1 in the gut were not present in dsRNA-treated schistosomula (Fig. 8), thus confirming the efficiency of knockdown.

## Discussion

Our focus on the subfamily M28B metalloproteases was guided by the fact that there was a complete lack of knowledge about these types of enzymes in parasitic platyhelminths. In mammals, this protease group contains several paralogs and splice variants with diverse and/or unknown functions. Their unique physiological roles remain unknown, as studies are complicated by their possible redundant roles or poor characterization. The primary expression sites of HsGCP2 under physiological conditions include the nervous system (astrocytes and Schwann cells), kidney (proximal tubules), and small intestine (jejunal brush border membranes). Several other studies have also pointed towards a broader GCP2 expression profile in humans, including lacrimal glands, heart, pancreas, bladder, skin, breast, liver, lung, colon, testis, etc., although expression levels in the latter tissues are thought to be significantly lower [18, 19]. Other human paralogs are localized in nervous tissue [24, 25] or with intestine-restricted expression and activity involved in protein/peptide degradation and absorption in the digestive system [15]. Contrary to mammals and many higher organisms, trematodes possess only a single gene encoding M28B subfamily orthologs. Their sequence alignment revealed the presence of all crucial conserved amino acid residues and domains that are essential for enzymatic function [15, 17, 57–59]. Nevertheless, the transmembrane domain and signal intracellular domain present in human GCP2 are absent from SmM28B and FhM28B orthologs, indicating that their intracellular function, or an alternative route of secretion [60, 61], are missing.

Our primary intention in investigating this group of enzymes in trematodes was to use these basal organisms as represented by *S. mansoni* and *F. hepatica*. We also hypothesized that these enzymes could lead to a druggable target as their in silico predicted substrate preferences are unique. Our principal effort was focused on basic topics such as localization, physiological functions, and essentiality for parasite survival. The localization of SmM28B and FhM28B approximately mirrors the expression profile of the partially characterized mammalian counterparts such as human GCP2, GCP3, and NAALADase L. Both proteases, SmM28B and FhM28B, were detected in the parasite organ structures that functionally correspond to those in mammals. Expression observed in the salivary glands and gut corresponded with localization in the digestive system of *S*. *mansoni* male and adult *F*. *hepatica*, particularly in the esophageal glands and the gastrodermis. Interestingly, we did not detect much of a signal in the gut of *S*. *mansoni* females, in agreement with the data presented in the single-cell RNA-seq atlas database [12]. However, the reason for this disproportional expression remains unclear until its physiological role becomes known. Other apparent expression localization sites are the reproductory organs of both flukes: gonads (ovaries and testes), oviducts, vitelline cells, and consequently in the eggs. In mammals, expression occurs in the reproductory organs; however, particular roles have not been clearly elucidated [18, 19]. Based on our research, both flukes express M28B proteases most likely in the cerebral ganglia, which resembles the roles of GCP2 and GCP3 in mammalian brain tissue. Our study revealed that SmM28B is also expressed as anti-sense RNA in *S. mansoni* vitellaria and oviducts. Anti-sense transcripts (i.e. non-coding RNAs) often play a regulatory role in post-transcriptional processes in various organisms [62] and are present in schistosomes [46, 63, 64].

The effectiveness of in vitro RNAi gene knockdown between schistosomula and adult *S. mansoni* was significantly different; nevertheless, no measurable phenotypic manifestations were observed. The low success with gene knockdown (approximately 30%) in adult worms, even in long-term experiments when fresh media containing dsRNA were replenished every second day, compared to approximately 95% in schistosomula, may be attributed to the significant differences in body sizes and morphologies of these two stages and tissue accessibility [33]. Unlike our observations in *Caenorhabditis elegans* (Dvorak lab, unpublished data), we could not achieve any significant phenotypic impact in vitro through targeting these proteases. Based on previous data [33] and a relatively low level of expression, in vitro conditions may not be ideal to reveal distinct functions in digestive, reproductive, and neuromotor functions. Thus, we set up a pilot experiment using in vitro exposed newly transformed schistosomula, which, after 2.5 days of incubation, were subsequently injected into laboratory mice (C57BL/6) as published previously [65]. Comparable numbers of worms, eggs, and granuloma formations were recorded, and the size and shape of recovered worms did not differ statistically. No obvious deformations or alterations were observed in treated worms 7 weeks p.i. (not shown). To conclude, our data based on RNAi do not provide adequate information. We can only speculate since we could not rule out, for example, that the loss of an RNAi effect a few days after mice infections may have been sufficient to permit recovery from the impact of knockdown. When compared with the phenotypization of GCP2 KO laboratory mice, this would confirm that the presence of the enzyme in schistosomula was not crucial for survival, at least under laboratory conditions [25].

The unusual feature of the M28B protease subfamily is that it encompasses both aminopeptidases and carboxypeptidases. However, so far, little is known about their natural substrates. For example, HsGCP2 is highly restrictive as the enzyme specifically removes the C-terminal glutamate and with a low preference for methionine only from dipeptides but not peptides with three or more residues [55, 66]. As for longer peptides as substrates for HsGCP2, the C-terminal glutamate would only be cleaved if it is linked to the penultimate residue via its γ-carboxylate group [20, 67]. It should be noted that γ-linked peptide bonds are not abundant in proteins but exist, for example, in dietary folates, which are natural substrates for human GCP2. Not surprisingly then, only screenings against the dipeptidic library revealed positive hits for HsGCP2, while no substrates were identified using the aminopeptidase or tetradecapeptide libraries. Identification of the substrates of M28B metalloproteases from platyhelminth parasites *S. mansoni* and *F*. *hepatica* would therefore contribute to general knowledge of their physiological roles [14]. Furthermore, different substrate specificities, compared to human orthologs, would offer an advantage for the design of selective inhibitors. Unfortunately, our screens could not identify any substrates of the protease from *F*. *hepatica*, yet the substrate specificity for HsGCP2 was consistent with known substrate preferences (Fig. 4) [60]. Several hypotheses can be offered to address this issue. First, although trematode orthologs possess all critical residues required for their enzymatic activity, they might lack the intrinsic hydrolase activity, similar to the transferrin receptor, which also belongs to the M28B family. Alternatively, there is a small chance that our HEK293 expression system was not suitable for the production of fully active proteins. For example, despite the absence of predicted signal/transmembrane sequences, proteins need to go through the secretory pathway, need to be N-glycosylated to possess peptidase activity (similarly to HsGCP2; [68]), or require an unknown co-factor for full activity. However, we believe that the most plausible explanation is a very narrow specificity of the proteases and that a preferred substrate recognized by the enzymes may be missing from our libraries. Further studies will be required to address these issues in more detail.

## Conclusions

This work represents the first deep exploration and partial characterization of the M28B subfamily of trematode metalloproteases orthologous to human glutamate carboxypeptidase 2. The blood fluke *S. mansoni* and the liver fluke *F. hepatica* were both chosen as relatively well-established laboratory models because of their pathogenic importance, well-known genome, and body organizations. The study of the specific functions of these groups of proteases in higher organisms is complicated because of the presence of several paralogs in their genome. Platyhelminths have only a single gene encoding M28B metalloprotease, however, the lack of experimental data in non-mammalian species precludes any comparison. Sequences of both trematode proteases were fully annotated and their structures were predicted based on homology modeling. These metalloproteases were recombinantly expressed, purified, and partially characterized. RT-qPCR revealed a gene expression profile for all life stages of *S. mansoni*. RNAi silencing did not lead to any apparent phenotypic manifestations and revealed a non-essential role for schistosomula surviving under laboratory conditions. In situ hybridization and immunohistochemistry confirmed the almost identical distribution of metalloproteases within tissues of both adult trematodes. Their distribution in trematodes coincides with their localization in functionally similar organ structures in mammals. We therefore hypothesize that M28B might play similar basic and universal physiological roles as their mammalian orthologs. Therefore, these enzymes may serve as one of several suitable models for future studies that could reveal the real physiological roles of these metalloenzymes.

## Supplementary Information

**Additional file 1: Table S1.**List of primers used for: **a** PCR amplification of the coding sequence of trematode M28B, used later for cloning into pD221 donor vectors by the BP Gateway cloning protocol (Invitrogen). All expression plasmids featuring N-terminal purification tags were prepared by recombining the donor vectors and in-house expression destination vectors using the LR Gateway reaction mix. **b** PCR amplification to obtain enzymatically inactive SmM28B (E407M) and FhM28B (E413M) mutants by the Quick-change site-directed mutagenesis using corresponding expression plasmids as templates.

**Additional file 2: Figure S1.** Map and detailed linker views of the pDEST320 destination vector.

**Additional file 3: Table S2.** List of primers used for RT-qPCR analyses of SmM28B, SmCB1, and SmCOXI.

**Additional file 4: Table S3.** Gene-specific primers for PCR amplification of SmM28B (**a, b**) and FhM28B fragments in sizes of 459 (**a**), 495 (**b**), and 451 bp, respectively. As templates, they served first-strand cDNA synthesis of adults *S. mansoni* and *F. hepatica*. The PCR fragments were ligated into the pGEM-T Easy Vector (Promega) and cloned sequences were verified by DNA sequencing.

**Additional file 5: Table S4.** List of primer sequences employed for dsRNA synthesis. Double-stranded RNA (dsRNA) was synthesized by the use of sense and antisense primers of each target gene. mCherry and SmCB1.1 were used as controls for our gene of interest, SmM28B. The T7 DNA polymerase-binding motif is shown in bold characters.

**Additional file 6: Figure S2.** SDS-PAGE analysis of FhM28B purification. The purified HALO-FhM28B fusion was cleaved by TEV protease and FhM28B was sequentially purified using Streptactin (columns **1–5**) and Ni-NTA (columns **6–8**) affinity chromatography. Proteins were separated by reducing SDS-PAGE and stained by Coomassie Brilliant Blue R-250. Lanes: **1**, purified HALO-FhM28B; **2**, reaction after TEV cleavage; **3** and **4**, flow-through from Streptactin column; **5**, elution from Streptactin column; **6** to **8**, flow-through from Ni-NTA column. Fractions **6** through **8** were pooled, concentrated, and used for rabbit immunization.

**Additional file 7: Figure S3.** Localization of mRNA encoding M28B protein in the cerebral tissues of *S. mansoni* and *F. hepatica* adults. ISH-reaction of antisense DIG-labeled RNA probes designed to label M28B sense mRNA (protein-coding mRNA) with histological sections (5 µm) of *S. mansoni* adults and (7 µm) of *F. hepatica* adults (red). Columns represent the head part of **a** female *S. mansoni*, **b** male *S. mansoni*, and** c **adult *F. hepatica* with the focus on cerebral tissues. M28B peptidase was detected in cerebral ganglia (cg) of both sexes of *S. mansoni* and the neural ring (nr) of adult *F. hepatica*. SmM28B was not detected in the ventral sucker (vs) or the oral sucker (os). The scale bars represent 50 µm.

**Additional file 8: Figure S4.** Localization of anti-sense RNA encoding SmM28B in *S. mansoni* adult female. Sections (7 µm) of *S. mansoni* female (**a-c**) were probed with DIG-labeled RNA probes designed to label SmM28B anti-sense RNA (non-protein coding RNA). The probe hybridized with transcripts was visualized by tyramide amplification assay (red). The adult female was monitored in three parts: **a** an anterior part of the worm, **b** oviduct and ovary, and **c** vitellaria. All columns show a fluorescent red signal merged with differential interference contrast. Gene expression of anti-sense SmM28B was detected in a few cells of oviduct (o) and vitellaria (v). Anti-sense RNA of SmM28B was not detected in muscles (mu) and tegument (tg), gut (g), parenchyma (p), ovaria (ov). The scale bars represent 100 µm.

## Data Availability

All materials and data are contained within the manuscript and supplementary figures. Sequences have been deposited in the NCBI GenBank database as *S. mansoni* SmM28B (GenBank MZ456528) and as *F. hepatica* FhM28B (GenBank MZ456529).

## Abbreviations

AbD: Antibody diluent
AMC: 7-Amino-4-methylcoumarin
BSA: Bovine serum albumin
DEPC: Diethyl-pyrocarbonate
ESP: Excretory secretory products
FhM28B: *Fasciola hepatica* M28B
FOLH: Folate hydrolase
GCP2: Glutamate carboxypeptidase 2
GuHCl: Guanidine hydrochloride
HsGCP2: Human glutamate carboxypeptidase 2
HPLC: High-performence protein liquid chromatography
IBs: Inclusion bodies
IHC: Immunohistochemistry
ISH: RNA in situ hybridization
MSP-MS: Multiplex substrate profiling by mass spectrometry
NAAG: N-acetyl-aspartyl-glutamate
NAALADase: N-acetylated-alpha-linked acidic dipeptidase
Ni–NTA: Nickel-nitrilotriacetic acid
NMRI: Naval Medical Research Institute
NTS: New transformed schistosomula
ORFs: Open reading frames
PBS: Phosphate-buffered saline
PCR: Polymerase chain reaction
PSMA: Prostate-specific membrane antigen
PZQ: Praziquantel
RT: Room temperature
RT-PCR: Reverse transcription-PCR
SDS-PAGE: Sodium dodecyl sulfate polyacrylamide gel electrophoresis
SmCB1.1: *Schistosoma mansoni* Cathepsin B1.1
SmCOX I: *Schistosoma mansoni* cytochrome C oxidase I
SmM28B: *Schistosoma mansoni* M28B
SSC: Saline-sodium citrate buffer
TCBZ: Triclabendazole
TSA: Tyramide signal amplification
