# Supplementary material for: Characterization of glutamate carboxypeptidase 2 orthologs in trematodes
Source: Parasit Vectors. 2022 Dec 20;15:480. doi: 10.1186/s13071-022-05556-5 (PMC9768917; doi:10.1186/s13071-022-05556-5)
Supplement: Supplementary file 1 — Additional file 1: Table S1.List of primers used for: a PCR amplification of the coding sequence of trematode M28B, used later for cloning into pD221 donor vectors by the BP Gateway cloning protocol (Invitrogen). All expression plasmids featuring N-terminal purification tags were prepared by recombining the donor vectors and in-house expression destination vectors using the LR Gateway reaction mix. b PCR amplification to obtain enzymatically inactive SmM28B (E407M) and FhM28B (E413M) mutants by the Quick-change site-directed mutagenesis using corresponding expression plasmids as templates. [file 13071_2022_5556_MOESM1_ESM.pdf]

Primer	Sequence	Use
**SmM28B_GTWf**	GAGAACCTGTACTTCCAGTCTAACATGTGGCAAGAAATATCACAAAATTTG	Forward primer; SmGCPII into pD221
**SmM28B _GTWr**	GGGGACCACTTTGTACAAGAAAGCTGGGTTATTATAATGCTAATGAAAAATCAGTTAAAC	Reverse primer; SmGCPII into pD221
**FhM28B_GTWf**	GAGAACCTGTACTTCCAGTCTAACATGATGTCAACTGACTGTTCAGATTGG	Forward primer; FhGCPII into pD221
**FhM28B _GTWr**	GGGGACCACTTTGTACAAGAAAGCTGGGTTATTATATCACACAAAACCCGTCAAC	Reverse primer; FhGCPII into pD221
**SmM28B_E407M_F**	GGATCCTGGGACGGTatGGAATTCTCTGTTTTG	mutagenesis
**SmM28B_E407M_R**	CAAAACAGAGAATTCCatACCGTCCCAGGATCC	mutagenesis
**FhM28B_E413M_F**	CCAGTTGGGATGGTatGGAGTTGTCATTGC	mutagenesis
**FhM28B_E413M_R**	GCAATGACAACTCCatACCATCCCAACTGG	mutagenesis
