# Supplementary material for: Characterization of glutamate carboxypeptidase 2 orthologs in trematodes
Source: Parasit Vectors. 2022 Dec 20;15:480. doi: 10.1186/s13071-022-05556-5 (PMC9768917; doi:10.1186/s13071-022-05556-5)
Supplement: Supplementary file 2 — Additional file 2: Figure S1. Map and detailed linker views of the pDEST320 destination vector. [file 13071_2022_5556_MOESM2_ESM.pdf]

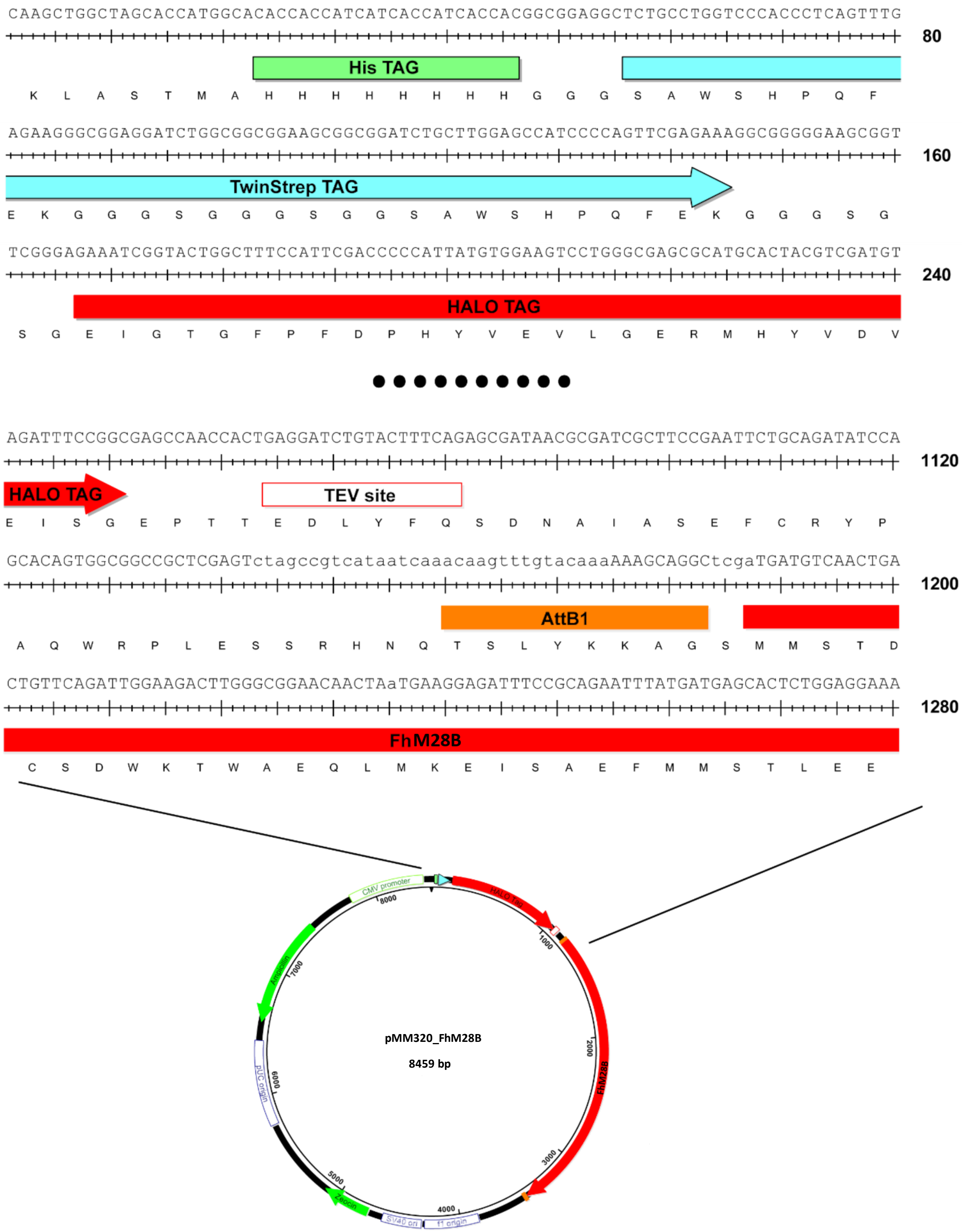

CAAGCTGGCTAGCACCATGGCACACCACCATCATCACCATCACCACGGCGGAGGCTCTGCCTGGTCCCACCCTCAGTTTG
80
His TAG
K L A S T M A H H H H H H H H G G G S A W S H P Q F
AGAAGGGCGGAGGATCTGGCGGCGGAAGCGGCGGATCTGCTTGGAGCCATCCCCAGTTCGAGAAAGGCGGGGGAAGCGGT
160
TwinStrep TAG
E K G G G S G G G S G G S A W S H P Q F E K G G G S G
TCGGGAGAAATCGGTACTGGCTTTCCATTCGACCCCCATTATGTGGAAGTCCTGGGCGAGCGCATGCACTACGTCGATGT
240
HALO TAG
S G E I G T G F P F D P H Y V E V L G E R M H Y V D V
AGATTTCCGGCGAGCCAACCACTGAGGATCTGTACTTTCAGAGCGATAACGCGATCGCTTCCGAATTCTGCAGATATCCA
1120
HALO TAG
TEV site
E I S G E P T T E D L Y F Q S D N A I A S E F C R Y P
GCACAGTGGCGGCCGCTCGAGTctagccgtcataatcaaacaagtttgtacaaaAAAGCAGGCtcgaTGATGTCAACTGA
1200
AttB1
A Q W R P L E S S R H N Q T S L Y K K A G S M M S T D
CTGTTCAGATTGGAAGACTTGGGCGGAACAACTAaTGAAGGAGATTTCCGCAGAATTTATGATGAGCACTCTGGAGGAAA
1280
FhM28B
C S D W K T W A E Q L M K E I S A E F M M S T L E E
CMV promoter
8000
HALO Tag
1000
Ampicillin
7000
pMM320_FhM28B
8459 bp
2000
FhM28B
pUC origin
6000
3000
5000
Zeocin
4000
SV40 ori
f1 origin
