# Supplementary material for: Characterization of glutamate carboxypeptidase 2 orthologs in trematodes
Source: Parasit Vectors. 2022 Dec 20;15:480. doi: 10.1186/s13071-022-05556-5 (PMC9768917; doi:10.1186/s13071-022-05556-5)
Supplement: Supplementary file 3 — Additional file 3: Table S2. List of primers used for RT-qPCR analyses of SmM28B, SmCB1, and SmCOXI. [file 13071_2022_5556_MOESM3_ESM.pdf]

Primer	Sequence	Use
**M28B_SM_qPCRf**	TCTTGGTTCTTGGGATGGAG	Forward primer
**M28B_SM_qPCRr**	TCGGTTCGAGCTGAAAAGTT	Reverse primer
**CB1.1_SM_qPCRf**	ACTTGGTGGGCACGCTATAC	Forward primer
**CB1.1_SM_qPCRr**	TAATTCGACCGGCTGTTACC	Reverse primer
**COXI_SM_qPCRf**	TACGGTTGGTGGTGTCACAG	Forward primer
**COXI_SM_qPCRr**	ACGGCCATCACCATACTAGC	Reverse primer
