# Supplementary material for: Characterization of glutamate carboxypeptidase 2 orthologs in trematodes
Source: Parasit Vectors. 2022 Dec 20;15:480. doi: 10.1186/s13071-022-05556-5 (PMC9768917; doi:10.1186/s13071-022-05556-5)
Supplement: Supplementary file 4 — Additional file 4: Table S3. Gene-specific primers for PCR amplification of SmM28B (a, b) and FhM28B fragments in sizes of 459 (a), 495 (b), and 451 bp, respectively. As templates, they served first-strand cDNA synthesis of adults S. mansoni and F. hepatica. The PCR fragments were ligated into the pGEM-T Easy Vector (Promega) and cloned sequences were verified by DNA sequencing. [file 13071_2022_5556_MOESM4_ESM.pdf]

Primer	Sequence	Use
**M28B_SM_ISf (a)**	GTGGCAACCCTCACTGTTCT	Forward primer
**M28B_SM_ISr (a)**	GAGTTCCGTTGCCCAGTCTA	Reverse primer
**M28B_SM_ISf (b)**	TCCTGGCGCTCTTATTCTGT	Forward primer
**M28B_SM_ISr (b)**	CAAGCACCTTGTACCCATGA	Reverse primer
**M28B_FH_ISf**	ACATCCAGTTGGCAGTGATG	Forward primer
**M28B_FH_ISr**	CTGTCTTGTGCCCTGTGAGA	Reverse primer
