# Supplementary material for: Characterization of glutamate carboxypeptidase 2 orthologs in trematodes
Source: Parasit Vectors. 2022 Dec 20;15:480. doi: 10.1186/s13071-022-05556-5 (PMC9768917; doi:10.1186/s13071-022-05556-5)
Supplement: Supplementary file 5 — Additional file 5: Table S4. List of primer sequences employed for dsRNA synthesis. Double-stranded RNA (dsRNA) was synthesized by the use of sense and antisense primers of each target gene. mCherry and SmCB1.1 were used as controls for our gene of interest, SmM28B. The T7 DNA polymerase-binding motif is shown in bold characters. [file 13071_2022_5556_MOESM5_ESM.pdf]

Target gene	Amplicon size (bp)	Primers for dsRNA synthesis	Sequence
**mCherry**	521	Cherry-T7-s	**TAATACGACTCACTATAGGG**ATGGTGAGCAAGGGCGAGGAG
		Cherry-as	TTACTTGTACAGCTCGTCC
		Cherry-s	ATGGTGAGCAAGGGCGAGGAG
		Cherry-T7-as	**TAATACGACTCACTATAGGG**TTACTTGTACAGCTCGTCC
**SmCB 1.1**	526	SmCB1-T7-s	**AAGTAATACGACTCACTATAGGG**ATGCTCACATCTATTTTGTGTATTGC
		SmCB1-as	CAATACCTTCCTTCACCCAGTAATC
		SmCB1-s	ATGCTCACATCTATTTTGTGTATTGC
		SmCB1-T7-as	**AAGTAATACGACTCACTATAGGG**CAATACCTTCCTTCACCCAGTAATC
**SmM28B**	483	SmM28b-T7-s	**TAATACGACTCACTATAGGG**TATCGTTTGGGGATTCCATCAACAT
		SmM28b-as	ATGATTTTTCACATTGACCAGCAGC
		SmM28b-s	TATCGTTTGGGGATTCCATCAACAT
		SmM28b-T7-as	**TAATACGACTCACTATAGGG**ATGATTTTTCACATTGACCAGCAGC
