# Supplementary figures and images for: Characterization of glutamate carboxypeptidase 2 orthologs in trematodes
Source: Parasit Vectors. 2022 Dec 20;15:480. doi: 10.1186/s13071-022-05556-5 (PMC9768917; doi:10.1186/s13071-022-05556-5)

(kDa) M 1 2 3 4 5 6 7 8

130

100

70

55

35

25

← HALO- FhM28B

← FhM28B

← HALO

← TEV

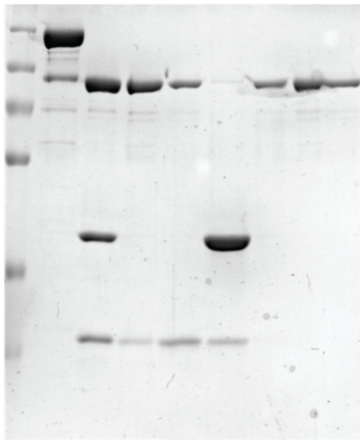

Supplement: Supplementary file 6 — Additional file 6: Figure S2. SDS-PAGE analysis of FhM28B purification. The purified HALO-FhM28B fusion was cleaved by TEV protease and FhM28B was sequentially purified using Streptactin (columns 1–5) and Ni-NTA (columns 6–8) affinity chromatography. Proteins were separated by reducing SDS-PAGE and stained by Coomassie Brilliant Blue R-250. Lanes: 1, purified HALO-FhM28B; 2, reaction after TEV cleavage; 3 and 4, flow-through from Streptactin column; 5, elution from Streptactin column; 6 to 8, flow-through from Ni-NTA column. Fractions 6 through 8 were pooled, concentrated, and used for rabbit immunization. [file 13071_2022_5556_MOESM6_ESM.pdf]

**a**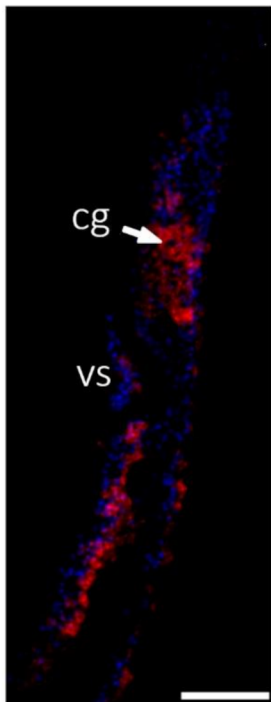**b**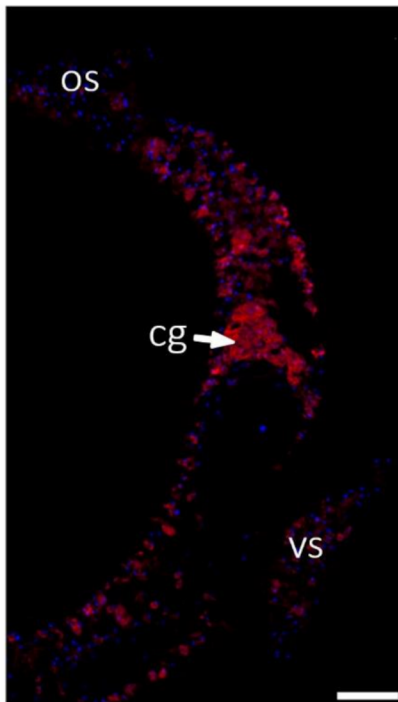**c**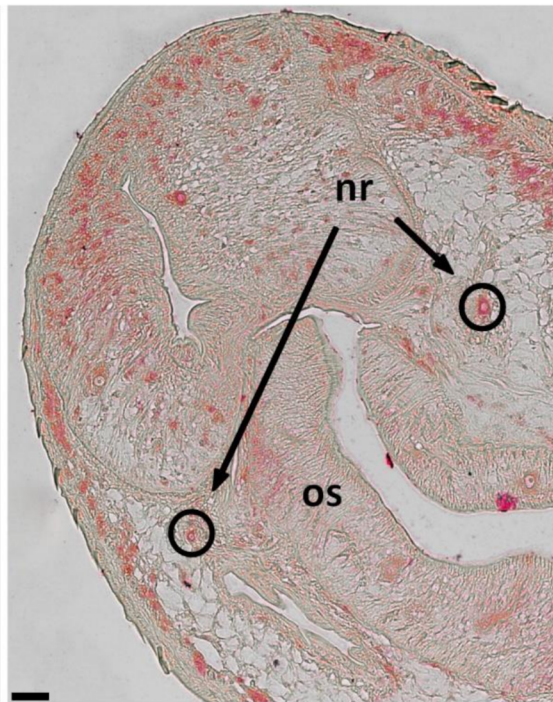

Supplement: Supplementary file 7 — Additional file 7: Figure S3. Localization of mRNA encoding M28B protein in the cerebral tissues of S. mansoni and F. hepatica adults. ISH-reaction of antisense DIG-labeled RNA probes designed to label M28B sense mRNA (protein-coding mRNA) with histological sections (5 µm) of S. mansoni adults and (7 µm) of F. hepatica adults (red). Columns represent the head part of a female S. mansoni, b male S. mansoni, and c adult F. hepatica with the focus on cerebral tissues. M28B peptidase was detected in cerebral ganglia (cg) of both sexes of S. mansoni and the neural ring (nr) of adult F. hepatica. SmM28B was not detected in the ventral sucker (vs) or the oral sucker (os). The scale bars represent 50 µm. [file 13071_2022_5556_MOESM7_ESM.pdf]

**a**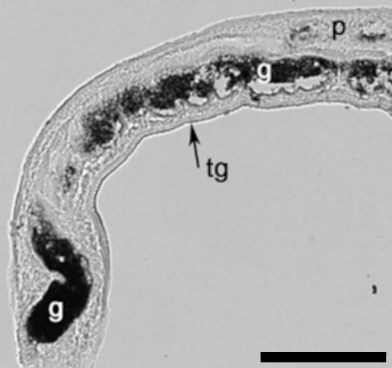**b**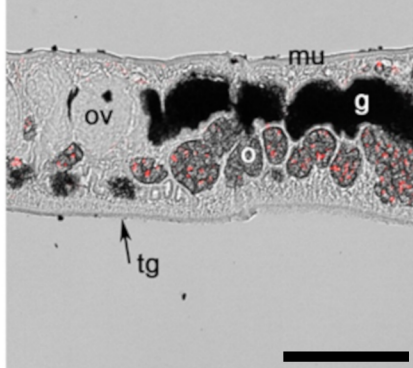**c**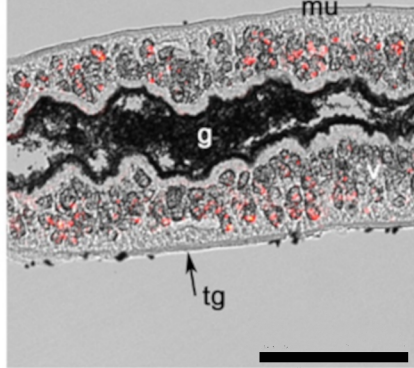

Supplement: Supplementary file 8 — Additional file 8: Figure S4. Localization of anti-sense RNA encoding SmM28B in S. mansoni adult female. Sections (7 µm) of S. mansoni female (a-c) were probed with DIG-labeled RNA probes designed to label SmM28B anti-sense RNA (non-protein coding RNA). The probe hybridized with transcripts was visualized by tyramide amplification assay (red). The adult female was monitored in three parts: a an anterior part of the worm, b oviduct and ovary, and c vitellaria. All columns show a fluorescent red signal merged with differential interference contrast. Gene expression of anti-sense SmM28B was detected in a few cells of oviduct (o) and vitellaria (v). Anti-sense RNA of SmM28B was not detected in muscles (mu) and tegument (tg), gut (g), parenchyma (p), ovaria (ov). The scale bars represent 100 µm. [file 13071_2022_5556_MOESM8_ESM.pdf]
